# Minor ailments, major problems: a critical appraisal of Rafferty et al. (2017)

**DOI:** 10.1186/s12962-018-0160-5

**Published:** 2018-11-19

**Authors:** Rudy Zimmer

**Affiliations:** Department of Community Health Sciences, The University of Calgary, Calgary, AB Canada

**Keywords:** Canada, Pharmacist, Pharmaceutical services, Physician, Nurse practitioners, General practice, Primary health care, Health policy, Conflict of interest, Economics

With increasing pressure to commercialize primary care within the larger publicly-funded provincial health care systems of Canada, bureaucrats and retail operators seem eager to show that new initiatives are worth public spending. A recent article by Rafferty and colleagues [1] funded by the Canadian Foundation for Pharmacy attempts to justify the value of Saskatchewan’s “Pharmacists Prescribing for Minor Ailments” or PPMA service in a study that is flawed in design, assumptions, inputs and thus conclusions. Though the authors state that such programs “aim to improve the efficiency of care [and] reduce physician visits” [1], recent outcomes research into other pharmacy services in Canada suggests otherwise [2, 3]. Depending on the level of clinical expectations, quality assurance research also calls into question the effectiveness of long-standing PPMA services in the United Kingdom [4]. Rafferty and colleagues have assumed that the PPMA service is safe, effective and efficient in Canada without providing any direct evidence to support these assumptions. Instead, they proceed with assessing “costs and savings” of a service that may cause more harm than good (e.g., promoting more prescription therapy for minor illnesses). A formal outcome evaluation using only primary data would have been a better starting point, expanding on the small convenience sample done earlier by Mansell and colleagues [5].

While the authors describe this study as an “economic impact analysis” or EIA, the actual design is not clear. It is not a formal EIA, which determines the financial impact of a program or institution on a regional or local economy [6, 7]. If the authors assumed that the safety and effectiveness of addressing minor illnesses using the PPMA service was the same as that of the alternative scenario (i.e., a modeled combination of patients seeing a doctor, treating themselves with over-the-counter (OTC) remedies, and doing nothing), then the design is a cost-minimization analysis (CMA) [8]. The “benefits” described by the authors are not health benefits monetized in a cost-benefit analysis (CBA) [8], but rather as operational and patient convenience “benefits” expressed as “savings”. True health benefits would be very limited, since the PPMA program is addressing “self-limited” conditions that often resolve by doing nothing. Savings cannot be assumed, since this PPMA program does not replace the status quo, but adds to it without a corresponding restriction on patients’ use of medical care. If there are no proven health benefits or real savings using this fee-for-service payment scheme with no ceiling, then calculating a return on investment (ROI) may also be meaningless for treating minor illnesses. Finally, the introduction of publicly-funded pharmacy services across Canada has been used as a vehicle for provincial governments to simultaneously reduce hidden drug “rebate” payments to retail pharmacies from drug manufacturers that have driven generic drug prices in Canada higher compared with similar countries [9]. The transfer of these previous rebates to the funding of pharmacy services was not included or analyzed. Are we simply moving the same public money around without really saving it?

Several inputs used in the economic models were biased in favor of the PPMA scenario. In the public payer model for example, the cost of family physician (GP) visits for minor illnesses was inflated by using a so-called “exploratory GP appointment” costing $66.40 [1] that does not exist in the Saskatchewan payment schedule for physicians [10]. The authors appear to be using a “3B” or “complete assessment” fee of $66.40, which would not be used for any GP visit to address minor illnesses without triggering a future billing audit. The appropriate input should have been a “5B” or $33.80 fee, which is for a “partial assessment or subsequent visit” that includes addressing more than one minor as well as major problems in one clinical encounter. In my opinion, the authors’ assumption regarding physician billing rules for publicly-funded medical services should have been validated by contacting the Ministry of Health or the Saskatchewan Medical Association. Pharmacists charge $18 per prescription per different minor ailment that may be billed in an additive fashion [11], which would exceed a doctor’s fee if both providers addressed two or more minor illnesses on the same day. Whereas a physician is not pressured to provide prescription drugs for every minor condition, a pharmacist must prescribe a drug to get paid. Thus, the PPMA may increase the incidents of adverse drug reactions that physicians would then need to address through additional GP visits. Emergency room (ER) visits are also costed using an average estimate, which averages minor visits with major trauma care and other expensive interventions. The input should have been at the low end of the cost range to reflect the true cost of a minor ER visit. Overall, the authors underestimated direct costs for the PPMA, and overestimate those for the alternative scenario.

In the societal scenario, the negative impact of retail pharmacy services must be considered, in the context of our publicly-funded health care being part of Canada’s national identity codified in legislation (e.g., the Canada Health Act) [12] or in our historical figures (e.g., Tommy Douglas as the father of modern Medicare) [13]. Any initiatives shifting health care delivery away from non-commercial clinical environments to market-driven retail spaces would need to be counted as a negative cost to our sense of identity. Less than 20% of community pharmacies in Canada are truly independent with the majority owned or strictly controlled through franchising agreements by large for-profit companies [14], which must put the private interests of their owners and shareholders ahead of the public interest [15].

Other biased societal costs include the following examples. Time waiting to see the doctor is equally costed to the time of the medical examination, rather than counting time with the doctor as being more beneficial to waiting. Also, monetization of waiting in the ER uses an average Canadian income to calculate costs, when it is established that patients of lower socioeconomic status will wait for hours in the ER to address minor ailments while others won’t [16]. Pharmacy wait times are optimistic, and would increase as more patients seek pharmacist advice for minor ailments. There may be additional indirect costs in seeking advice within a retail environment, where the patient may be encouraged to purchase additional unproven health products compared with seeking medical attention in a neutral clinic space. Finally, it is impossible to extrapolate out 5 years regarding health care systems that are in constant flux. The introduction of prescribing nurses and physician assistants may reduce demand for PPMA services in the future.

Independent higher quality evaluations of new services provided by community-based retail pharmacies in Canada are badly needed, as the current body of knowledge has an established publication bias toward positive findings [17].

## Abbreviations

PPMA: pharmacists prescribing for minor ailments
EIA: economic impact analysis
OTC: over-the-counter
CMA: cost-minimization analysis
CBA: cost–benefit analysis
ROI: return on investment
